# Audit on Hysterectomies Done in a Tertiary Care Center: Addressing the Dilemma on Retaining the Uterus From Womb to Tomb

**DOI:** 10.7759/cureus.80081

**Published:** 2025-03-05

**Authors:** Dhaneshwor Phijam, Wangol Kiyam, Yohen Nandeibam, Dipenty Lairenjam, Gyaneshowri Laishram

**Affiliations:** 1 Department of Obstetrics and Gynaecology, Shija Academy of Health Sciences, Imphal, IND; 2 Department of Pathology, Shija Academy of Health Sciences, Imphal, IND

**Keywords:** clinico-pathological correlation, histopathological concordance, hysterectomy, prevalence, retaining the uterus

## Abstract

Introduction

This study attempts to determine whether hysterectomies in tertiary care centers are avoidable and explores the possibility of retaining all uteri from womb to tomb.

Objectives

The objective of this study is to determine the incidence of women less than 35 years of age, bilateral salpingo-oophorectomy in women less than 40 years of age, insufficient documentation of indication or prior conservative treatment for the symptoms, discrepancy between indication for surgery and histopathological examination reports, and associated with severe postoperative morbidity or mortality.

Methods

This is a retrospective cohort study reviewing medical records of patients admitted for hysterectomy for various indications in a tertiary center in north-east India.

Results

Among 201 hysterectomies, the most common indication was abnormal uterine bleeding with the most common histopathological finding being leiomyoma; 3.5% of women were under 35 years, 32 women were below 40 years of which only one (0.5%) had bilateral salpingo-oophorectomy. All case records mentioned the indication for surgery, though lacking details of prior treatments. The concordance rate between surgeons’ indication and final histopathological examination reports was high, with Spearman’s correlation coefficient of 0.82.

Conclusion

Though all attempts should be made toward conserving the uterus with benign pathologies, especially those with abnormal uterine bleeding, many of the hysterectomies in tertiary centers are likely to be unavoidable, as most of the patients are secondary referrals from other centers where conservative management may have failed.

## Introduction

Hysterectomy is the most common surgery in women, with a lifetime risk of undergoing the procedure ranging from one in three to one in five in developed countries based on population-based retrospective cohort studies [1,2]. Data on prevalence are, however, limited in developing countries.

Concerns have been raised regarding the high prevalence of hysterectomy in young women well away from menopause, especially among those with health insurance or welfare schemes [3].

National Family Health Survey 4 in India, revealed a high prevalence of hysterectomy for indications, such as abnormal uterine bleeding (AUB), where conservative nonsurgical methods can be used. An interesting finding in the same survey was a higher prevalence of hysterectomy in economically advanced states, such as Andhra Pradesh, compared to the northeastern states of India. A trend of higher rates of hysterectomy in private hospitals compared to government hospitals was noted almost all over India, while the northeastern states showed an opposite trend [4]. We conducted a retrospective audit in a tertiary care center to ascertain whether hysterectomies in our center were avoidable.

## Materials and methods

We conducted a retrospective review of in-patient records of patients admitted for hysterectomy from 1st January 2021 to 31st December 2023, in a tertiary care center in Northeast India.

Inclusion criteria

Records of all women undergoing hysterectomy through laparoscopic, abdominal or vaginal route were included in the study. Hysterectomy for any indication and at any age was included in the study.

Exclusion criteria

Women who underwent emergency hysterectomy or obstetric hysterectomy were excluded from the study.

Primary outcome

We reviewed the histopathological examination (HPE) reports of the surgical specimen following hysterectomy to find concordance between indication for surgery and HPE report.

Secondary outcome

We also looked at the incidence of women less than 35 years of age, women less than 40 years of age who had bilateral salpingo-oophorectomy (BSO), insufficient documentation in the medical records of indication for surgery or prior treatments and incidence of severe postoperative morbidity or mortality.

Data entry

One presenting clinical symptom and the most probable clinical diagnosis were considered and recorded as the indication for the surgery. Age of the women at the time of hysterectomy, her parity, and the route of surgery were noted. All intra-operative or immediate postoperative complications were included in the clinical data sheet. HPE reports following the surgery were reviewed. Indications for hysterectomy were compared with histopathological data. We also screened the medical records of these women for any subsequent readmissions required due to surgical complications such as wound infection, trocar site hernia, or undetected urogenital tract injury.

Statistical analysis

Microsoft Excel software (Microsoft® Corp., Redmond, WA) was used for data entry, and statistical analysis on correlation was done using Spearman’s correlation coefficient on SPSS (IBM SPSS Statistics for Windows, IBM Corp., Version 29.0.2.0, Armonk, NY).

## Results

During the study period, 201 hysterectomies were done for various indications. There were two additional cases of emergency hysterectomy done for placenta previa and invasive mole, which have been excluded from the study.

Clinical indication and prior treatment for presenting symptoms

All the women operated were symptomatic, and their case records reviewed mentioned the clinical indications for hysterectomy and type of surgery planned whether laparoscopic, vaginal or abdominal, but failed to mention prior treatment for the presenting symptoms. Associated comorbidities and previous surgeries, however, were noted in all the records.

Age, parity, and bilateral salpingo-oophorectomy

Demographic data are summarized in Table 1. The majority of the patients were in the age group of 46-50 years of age (29.8%), followed by the 40-45 years age group (23.8%). Most of them were multiparous (81%). There were seven women under the age of 35 years who underwent hysterectomy but none of them had BSO. Twenty-five women between 36 and 39 years underwent hysterectomy, of whom only one had BSO. Five nulliparous women underwent hysterectomy, four of them with BSO. All of them were above 40 years of age. Indications included awareness of mass per abdomen with fibroid uterus among two postmenopausal women, and heavy menstrual blood loss (HMBL) in three perimenopausal women.

**Table 1:** Patient characteristics and clinical presentations

	No. of patients (%), N = 201
Age group (years)	
<35	7 (3.5)
36-39	25 (12.4)
40-45	48 (23.8)
46-50	60 (29.8)
51-55	34 (16.9)
56-60	11 (5.7)
>60	16 (7.6)
Parity	
0	5(2.6)
1	33(16.4)
2	126(62.6)
>2	37(18.4)
Clinical presentation	
Menorrhagia with/without dysmenorrhea or dyspareunia	72 (35.9)
Dysmenorrhea/dyspareunia/pelvic pain	55 (27.4)
Post-menopausal bleeding	15 (7.5)
Mass per abdomen	30 (14.9)
Mass per vagina	17 (8.5)
Pain abdomen	4 (2.0)
White discharge per vagina	7 (3.5)
Low backache	1 (0.5)

Clinical indication for hysterectomy

Clinical indication for surgery was mentioned in the in-patient record as well in the surgical consent forms. A total of 158 (78.6%) patients underwent hysterectomy for indications included under PALM COEIN (polyp, adenomyosis, leiomyoma, malignancy, coagulopathy, ovulatory dysfunction, endometrial, iatrogenic, and not yet classified) classification (Table 2). Most common indication for hysterectomy under PALM COEIN was leiomyoma (119, 59.2%) followed by adenomyosis (20, 10%). There was no hysterectomy done for Coagulopathy, Iatrogenic, and Not Yet Classified groups.

**Table 2:** PALM COEIN and non-PALM COEIN indications for hysterectomy PALM COEIN - polyp, adenomyosis, leiomyoma, malignancy, coagulopathy, ovulatory dysfunction, endometrial, iatrogenic, and not yet classified

Indications	Number (%)	Total (%)
PALM COEIN indications	-	158 (78.6)
Polyp (leiomyomatous polyp)	1 (0.5)	-
Adenomyosis	20 (10.0)	-
Leiomyoma	119 (59.2)	-
Malignancy/premalignant endometrial malignancy hyperplasia	1 (0.5), 4 (2.0)	-
Coagulopathy	0	-
Ovulatory dysfunction	4 (2.0)	-
Endometrial	9	-
Iatrogenic	0	-
Not yet classified	0	-
Non-PALM COEIN indications	-	43 (21.39)
Pelvic organ prolapses	17 (8.5)	-
Cervical polyp	1 (0.5)	-
Cervical intraepithelial neoplasia	2 (1.0)	-
Carcinoma cervix	2 (1.0)	-
Ovarian cyst	19 (9.5)	-
Ovarian carcinoma	2 (1.0)	-

There were 43 (21.39%) women who underwent hysterectomy for various indications not classified under PALM COEIN (Table 2), most common indication being benign ovarian cysts (19, 9.5%). There were five (2.5%) cases of hysterectomy done for malignant and six (3%) for premalignant indications.

Route for hysterectomy

Total laparoscopic route for hysterectomy (174, 86.5%) was the most common, followed by vaginal (17, 8.5%), and abdominal route (10, 5.0%). Hysterectomy with bilateral salpingectomy or unilateral salpingectomy was done in 157 (78.1%), with unilateral salphingo-oophorectomy in 10 (5.0%), and with BSO in 18 (9.0%) women.

Histopathological concordance

Based on HPE, chronic cervicitis was most commonly reported in the cervix (70, 34.8%), and was the only significant finding in those operated for indications such as AUB - ovulatory and pelvic organ prolapse. Endometrial hyperplasia was reported in 16 women (16, 8.0%), of which only four were preoperatively diagnosed. Leiomyoma was the most frequently reported HPE finding. However, no case of leiomyosarcoma was detected. The histopathological findings are summarized in Table 3.

**Table 3:** Histopathological findings

Specimen	Histopathology	No. of patients(%, N = 201)
Cervix	Chronic cervicitis	70 (34.8)
	Cervical intraepithelial neoplasia	4
	Carcinoma cervix	2
	Benign cervical polyp	3
Endometrium	Polyp	11
	Endometritis	1
	Hyperplasia	16
	Carcinoma	1
	Atrophic	10 (5.0)
Myometrium	Leiomyoma	121 (60.2)
	Fibroid polyp	1
	Adenomyosis	32 (16.0%)
Fallopian tubes	Cysts	3 (1.5)
	Hydrosalpinx	2
	Salpingitis	2
Ovaries	Benign ovarian cyst	37 (18.4)
	Cancer	2
	Fibroma	1

On HPE, except for adenomyosis, the clinical diagnosis matched with the histopathology in most cases (Table 4). Spearman’s rho correlation coefficient was 0.82 (level of significance 0.01, two-tailed), suggestive of significant concordance between preoperative indication for surgery and HPE.

**Table 4:** Concordance between preoperative indication and histopathological diagnosis

Pathology	No. of preoperative diagnosis	No. of histopathological diagnosis	No. of correctly diagnosed	% Concordance
Leiomyomatous polyp	1	1	1	100%
Adenomyosis	20	32	11	55%
Leiomyoma	119	121	109	91.6%
Abnormal uterine bleeding - ovulatory	4	Not applicable	-	-
Endometrial polyp	9	11	9	100%
Pelvic organ prolapses	17	Not applicable	-	-
Cervical polyp	1	3	1	100%
Benign ovarian cyst	19	37	19	100%
Cervical intraepithelial neoplasia	2	4	2	100%
Endometrial hyperplasia	4	16	4	100%
Carcinoma cervix	2	2	2	100%
Carcinoma endometrium	1	1	1	100%
Ovarian carcinoma	2	2	2	100%
Total	201	-	-	-

Out of 32 histopathological reports of adenomyosis, only 11 cases were correctly diagnosed preoperatively. Only four cases of endometrial hyperplasia, out of 16 histopathological reports, were known preoperatively. Out of four cervical intraepithelial neoplasia (CIN), only two were known preoperatively.

Postoperative morbidity

There were no records with immediate severe postoperative morbidity such as massive blood transfusions, admission to intensive care, re-laparotomy for visceral injury in the study period. Most patients were admitted on the day of surgery and discharged either on the day after surgery or the following day, thus requiring two to three days of hospital stay.

However, four women were readmitted for delayed complications following total laparoscopic hysterectomy. Two women had ureteric fistula detected on about the 10th postoperative day, of which one was managed by open and the other by laparoscopic ureteric reimplantation with psoas hitch. Two women reported with vaginal discharge about one week after the procedure and were found to have vault abscess, which was managed with oral broad-spectrum antibiotics and vault toileting. None of our patients had trocar site hernia.

## Discussion

Ours is a tertiary care center catering mostly to high-risk pregnancies and gynecological referrals from both public and private health care units within the state of Manipur and neighboring states of Mizoram, Nagaland, and Arunachal Pradesh in the northeastern part of India. Occasional cross-border patients from the neighboring country of Myanmar are also seen in our hospital. The number of live births ranges from 450 to 500 in a year. Additionally, we conduct about 60 to 70 hysterectomies in a year.

Since many of the patients were usually secondary referrals, we assumed that most of the hysterectomies would be unavoidable. This audit was an attempt toward this end.

Hysterectomy in women under 35 years

Hysterectomy at a young age, less than 35 years (3.5%) or BSO in those below 40 years (0.5%) was rarely practiced. Similar to reports published from other tertiary care centers and teaching hospitals, the majority of our patients were in the age group of 40 to 50 years (53.6%). Kasinathan and Nagulapally in a retrospective study to correlate histopathology with indication reported that the average age was 49.72 years and none below 45 years had BSO. The age range of women was between 37 and 65 years [5]. In a similar study to correlate histopathology and clinical indication for abdominal hysterectomy in a teaching hospital, Sultana et al. reported that 70% of the women were in the age group of 40 to 50 years, and 30% were below 40 years of age [6]. Further, Tiwana et al. also reported that the age group of women undergoing hysterectomy in a teaching hospital ranged from 22 to 85 years, but the mean age was 45 ± 9.2 years. Similar to ours, 46.4% of the women were between 41 and 50 years of age. However, 36% of the women reported were 40 years or under. In contrast to our audit, they included patients who underwent emergency and obstetric hysterectomies, which may explain the very young age [7]. Gupta et al. also reported hysterectomies in women in the age range of 20 to 80 years, though 51% of them were in the age range of 40 to 49 years. About 22% of the women operated were below 40 years of age in their study [8]. Wankhede and Dawande in a retrospective observational study on the histopathology following hysterectomy found that most hysterectomies were among women in the age group of 35 to 45 years (72%), and 12.7% were among those below 35 years of age. BSO was not addressed in the study [9].

Hysterectomy and parity

Parity was not reported in most of the studies. In our study, 97.4% of the women were parous. Arya et al. reported a similar number (98%) of parous women among those undergoing hysterectomy [10]. Only 2.6% of the hysterectomies were in nulliparous women; however, none of them were less than 40 years of age.

Indication for hysterectomy

The most common indication in our study was HMBL or menorrhagia at 35.9%, followed by pain symptoms such as dysmenorrhea, dyspareunia, pelvic pain, or abdominal pain at 29.4%; a trend similarly reflected in most published literature. Arya and Bhushan reported the most frequent presenting symptoms as excessive or irregular bleeding (62%), and pain in the abdomen 29% [10]. Sultana et al. reported 71% menorrhagia, 40% dysmenorrhea, and 28% pelvic pain among the presenting symptoms [6]. Shanmukhi et al. in a prospective observational study found that AUB was the most common indication for hysterectomy (50%) and followed by dysmenorrhea, dyspareunia, and pelvic pain (28%) [11]. Gothwal et al. retrospectively reviewed patient records and reported that among patients operated for AUB, structural lesions are most common with leiomyoma being reported in about 41% of their patients who underwent hysterectomy [12]. Our study showed that the percentage of patients operated for HMBL was much lower than elsewhere, suggesting the use of conservative managements and opting for surgical intervention when other methods have failed. However, this was not documented in the clinical case records. Our study being retrospective in nature, ascertaining this observation was not feasible. Interestingly Gupta et al. reported dysfunctional uterine bleeding (DUB) as clinical indication in only about 8% of the women operated, and their predominant indication was uterovaginal prolapse (40%) [8]. A general trend can be noted where there was a lack of conservative management for AUB. Many tertiary centers have reported the clinical indication as AUB in more than 50% of their hysterectomies.

Histopathological correlation

Among patients presenting with AUB, the most common indication for hysterectomy, under the PALM COEIN classification, was uterine leiomyoma (59%), which is also reflected similarly in other studies. Arya and Bhushan reported that 49% of their patients had leiomyoma uteri, which correlated with the 48% preoperative diagnosis of fibroid uterus, in those presenting with bleeding disorders [10]. Sultana et al. reported a high clinical correlation between their preoperative diagnosis of fibroid uterus (52%) among patients with AUB with 49% proven by histopathology [6]. Verma et al., in a retrospective analysis, found that around 10% of patients were operated for DUB. In contrast to the findings of previous authors, they found poor correlation between histopathology and clinical diagnosis. Histopathology revealed adenomyosis, endometrial hyperplasia, polyp and placental site trophoblastic neoplasm, which were missed preoperatively, in addition to cystic glandular hyperplasia. AUB-endometrium was thus the more common indication for hysterectomy in their study. Fibroid was an indication in about 25% of their cases, which correlated with the histopathological diagnosis [13]. Tiwana et al.’s audit showed that in 50% of the patients with a preoperative diagnosis of DUB, leiomyoma was the predominant histopathological finding. The audit also found many findings, such as endometrial hyperplasia and adenomyosis, which were missed preoperatively. In the age group between 30 and 40 years, 78% of the hysterectomies involved BSO in which there was no pathology in 98% of the ovaries, which also showed poor clinical correlation and a lack of indication for removal of the ovaries. Whereas in our study, we found that ovaries were conserved in almost all patients younger than 40 years [7]. In a prospective cohort study of patients undergoing hysterectomy, Shanmukhi et al. observed that leiomyoma was the most frequent HPE finding in those operated for AUB, and similar to our study, no patients were operated for indications such as coagulopathy, iatrogenic cause, or “not yet classified” [11].

The HPE reports were concordant with clinical diagnosis in 90.5% of cases (182/201). There was poor concordance in cases of adenomyosis. Out of 32 cases of adenomyosis as per HPE report, only 11 cases were diagnosed preoperatively, and another nine cases were overdiagnosed. Therefore, 67% of total cases of adenomyosis were missed in the preoperative diagnosis. A similar picture is reflected in our review of literature where adenomyosis is mentioned to be the pathology most commonly missed in the preoperative diagnosis [7,13]. This indicates the need to be aware of this entity, to avoid missing or overdiagnosing preoperatively.

There was no case of leiomyosarcoma in this study; as such, the incidence is very rare (0.13%) [14,15].

None of the histopathological reports showed unremarkable pathology, mostly justifying the indication of hysterectomy.

Route for hysterectomy

About 93% of our hysterectomies were minimally invasive either through laparoscopy or vaginal route. This may be explained by ours being a tertiary center with ready availability of expertise and equipment. A major deciding factor for this is the patient’s choice, after having been made aware of the risks, benefits, and costs involved in all alternatives. The most common type of procedure was total laparoscopic hysterectomy with bilateral salpingectomy. It is a practice in our center to perform opportunistic salpingectomy during hysterectomy. Majority of the hysterectomies were done for benign diseases compared to that of malignancy (97.5% vs. 2.5%).

Ovaries were conserved in most of our cases (88.5%). We emphasize the practice of conservation of ovaries during hysterectomy unless there is a clear indication of any pathology in the ovaries [13]. The deleterious effects of oophorectomy, such as increased risk of coronary artery disease, decline in cognitive functioning, and sexual functioning, as well as a decrease in bone mineral density, warrant conserving the ovaries. The risk of ovarian malignancy in such women is likely mitigated by our practice of opportunistic salpingectomy [16]. All the cases of hysterectomy except those done for uterovaginal prolapse showed significant HPE findings. Except for one case showing adenomyosis, no significant histopathology was detected in the uterine specimens operated for uterovaginal prolapse.

## Conclusions

Significant concordance was observed between indication for hysterectomy in a tertiary center and histopathological reports. In addition, none of the surgical specimens reported unremarkable pathology, justifying the decision to proceed with hysterectomy in most cases. Though attempts should be made to conserve the uterus for all benign indications, however, the procedure remains unavoidable. Heavy menstrual bleeding remains the most common indication for hysterectomy. This is also the indication where effective conservative management options are readily available. Most hysterectomies in tertiary centers are likely to be indicated surgeries having failed with other methods in referring units.
